# Changes in harm perceptions of e-cigarettes compared with cigarettes following the announcement of the disposable vape ban in Great Britain

**DOI:** 10.1093/ntr/ntag030

**Published:** 2026-02-12

**Authors:** Sarah E Jackson, Jamie Brown, Catherine Kimber, Katherine East, Emke Brazier, Sharon Cox

**Affiliations:** Department of Behavioural Science and Health, University College London, London, UK; Department of Behavioural Science and Health, University College London, London, UK; Behavioural Research UK, London, UK; Nicotine, Tobacco and Vaping Research Group, College of Health and Life Sciences, London South Bank University, London, UK; Department of Primary Care and Public Health, Brighton and Sussex Medical School, University of Brighton and University of Sussex, Brighton, UK; Department of Addictions, Institute of Psychiatry, Psychology and Neuroscience, King’s College London, London, UK; Nicotine, Tobacco and Vaping Research Group, College of Health and Life Sciences, London South Bank University, London, UK; Department of Behavioural Science and Health, University College London, London, UK; Behavioural Research UK, London, UK

**Keywords:** e-cigarettes, harm perceptions, risk perception, vaping policy, smoking cessation, disposable vapes

## Abstract

**Introduction:**

E-cigarettes (“vapes”) are less harmful than cigarettes and effective for smoking cessation. However, public perceptions of their relative harms have worsened over the past decade. In January 2024, the UK government announced a forthcoming ban on disposable e-cigarettes, which received extended media coverage. Concerns were raised that this could exacerbate negative harm perceptions.

**Methods:**

We conducted a repeat cross-sectional study using monthly data from the Smoking Toolkit Study (January 2022–June 2025, the month the ban was implemented). Segmented regression models assessed changes in relative harm perceptions of e-cigarettes versus cigarettes before and after the January 2024 announcement. Participants were 16 489 people (≥16 years) in Great Britain who smoked.

**Results:**

Trends in harm perceptions changed following the announcement of the ban on disposable e-cigarettes. Between January 2022 and January 2024, the proportion who believed e-cigarettes were less harmful declined (RR = 0.804 [95% CI = 0.764–0.846]), while those believing e-cigarettes were more (RR = 1.300 [1.226–1.379]) or equally harmful (RR = 1.078 [1.033–1.124]) increased, and the proportion unsure decreased (RR = 0.859 [0.805–0.917]). Following the policy announcement, the decline in less harmful perceptions and increase in more harmful perceptions both decelerated significantly (post-announcement trends: RR = 0.922 [0.806–1.055]; RR = 1.073 [0.939–1.227]). By June 2025, 18.7% [17.1%–20.5%] believed e-cigarettes were less harmful, 31.3% [29.1%–33.7%] more harmful, 37.5% [35.5%–39.6%] equally harmful, and 12.5% [10.9%–14.3%] were unsure.

**Conclusions:**

Concerns that the UK government’s 2024 vaping policy announcement would exacerbate worsening trends in negative harm perceptions of e-cigarettes among people who smoke appeared unfounded: the rate of deterioration in harm perceptions actually slowed significantly to June 2025. However, harm perceptions still declined, albeit more slowly, and a large proportion of people who smoke continue to hold misperceptions about the relative harms of e-cigarettes.

**Implications:**

Misperceptions about the relative harms of e-cigarettes remain common among people who smoke in Great Britain. The slowing of negative trends following the disposable ban announcement suggests the policy announcement did not worsen deteriorating harm perceptions as feared. Nonetheless, with only one in five adults who smoke recognizing that e-cigarettes are less harmful than cigarettes, clear communication from public health bodies, government, and via the media is needed to address ongoing misconceptions. Future research should assess whether implementation of the ban in June 2025 has different effects on harm perceptions.

## Introduction

Accurate public understanding of the relative harms of nicotine products is essential to support informed decision-making and effective public health strategies. E-cigarettes (“vapes”) are widely recognized by leading health organizations in the United Kingdom, including the Office for Health Improvement and Disparities, as substantially less harmful than combustible tobacco.^1^ However, public perceptions of e-cigarette harms have become increasingly misaligned with current evidence, with growing numbers of people believing that e-cigarettes are equally or more harmful than smoking.^2^^,^^3^ Smoking can be a difficult behavior to change,^4^ and so this misperception may further deter people who smoke from switching to e-cigarettes,^5^ undermining efforts to reduce smoking-related harm.

Disposable (also known as single-use) e-cigarettes are battery-operated hand-held devices designed to be used and then discarded once the liquid contained within the reservoir has run out. They are pre-filled with an often flavored and nicotine-containing liquid. As these products are single-use, they are cheaper to buy, which is one reason they have appealed to a wide range of users, including young people.^6^ In January 2024, the UK government announced a forthcoming ban on disposable e-cigarettes for environmental reasons under the Department for Environment, Food & Rural Affairs,^7^ which received extended media coverage.^8^ In March 2024, a broader range of tobacco control measures and vaping restrictions were also announced under a new Tobacco and Vapes Bill, to reduce appeal to young people and children.^9^ These include powers to regulate the way vaping products are manufactured, packaged, marketed, and sold. The disposable e-cigarette ban came into effect in June 2025. While the policy aimed to reduce youth uptake and environmental waste,^7^ concerns were raised about its potential to further distort public perceptions of e-cigarette harms, particularly among adults who smoke.^10–12^ Critics argued that against a backdrop of already poor perceptions, banning a widely used form of e-cigarette could unintentionally signal to the public that all e-cigarettes are inherently dangerous.^11^^,^^12^ Given the rapid succession of policy changes and extensive media attention, it is particularly important to understand how such policy announcements influence public beliefs, as these perceptions can affect smoking cessation behaviors^5^ and overall population health.

This study examined changes in the relative harm perceptions of e-cigarettes compared with cigarettes among people who smoke in Great Britain between January 2022 and June 2025, with a particular focus on how these trends shifted following the government’s January 2024 policy announcement. Specifically, we aimed to establish whether the announcement was associated with a significant change in the rate at which beliefs about e-cigarette harms were evolving.

## Materials and methods

### Design

We analyzed data from the Smoking Toolkit Study, an ongoing monthly cross-sectional survey of a representative sample in Great Britain.^13^^,^^14^ Each month, a new sample of approximately 2450 people aged ≥16 years is recruited using a hybrid of random probability and simple quota sampling. Data are collected via telephone interviews. Comparisons with other national surveys and sales data suggest that the survey provides nationally representative estimates for key sociodemographic characteristics and nicotine use behaviors.^13^^,^^15^

For these analyses, we used data collected between January 2022 (around 6 months after disposable e-cigarettes began gaining popularity^16^ and 2 years before the government’s announcement of the ban^7^) and June 2025 (the most recent data available at the time of analysis, and the month when the disposable e-cigarette ban was implemented).

### Measures

Smoking status was assessed by asking participants which of the following best applied to them: (a) I smoke cigarettes (including hand-rolled) every day; (b) I smoke cigarettes (including hand-rolled), but not every day; (c) I do not smoke cigarettes at all, but I do smoke tobacco of some kind (eg, pipe, cigar, or shisha); (d) I have stopped smoking completely in the last year; (e) I stopped smoking completely more than a year ago; (f) I have never been a smoker (ie, smoked for a year or more). Responses a-c were considered current smoking and included in the analytic sample.

Harm perceptions of e-cigarettes compared with cigarettes were assessed among those who reported current smoking with the question: “Compared to regular cigarettes, do you think electronic cigarettes are more, less, or equally harmful to health?” Response options were “more harmful,” “less harmful,” “equally harmful,” or “don’t know.” Responses were dummy coded for analysis to produce a separate variable for each harm perception (eg, more harmful vs. all other responses).

### Statistical analysis

Data were analyzed using R v.4.4.1. These analyses were not pre-registered and should be considered exploratory. The Smoking Toolkit Study uses raking to create survey weights that match the sample to the population in Great Britain.^14^ The following analyses were done on weighted data.

We used segmented regression to examine changes in monthly trends in each harm perception of e-cigarettes compared with cigarettes among people who smoke in Great Britain following the government’s announcement of new vaping policies. We analyzed data overall and stratified by vaping status (ie, distinguishing between participants who reported exclusive smoking vs. dual use).

Trends were modeled using log-binomial generalized additive models with the *mgcv* package in R. The secular trend was represented by a continuous variable for each month (January 2022 = 1 through June 2025 = 42), and the change in slope post-announcement was modeled with a second variable (coded 0 up to January 2024, then 1 to *n* for each month thereafter). To account for seasonal variation, we included a month-of-year variable (January = 1 to December = 12) modeled with a cyclic cubic spline. We assumed a linear trend in log-prevalence before the announcement, implying a stable proportional month-on-month change. Given the relatively short time series, we anticipated minimal differences between log-linear and linear models.

To aid interpretation, model coefficients were multiplied by 12 to express changes on an annual scale. Post-announcement trends were calculated by summing the pre-announcement and slope-change coefficients on the log scale and exponentiating the result to convert it to an annual risk ratio. Corresponding 95% confidence intervals (CIs) were derived by combining the uncertainties of both components, calculating the standard error, and applying the usual CI formula before exponentiating. Predicted values (via the *predict* function) were used to plot modeled trends alongside unmodeled data points for each harm perception.

## Results

A total of 100 469 people aged ≥16 years were surveyed in Great Britain between January 2022 and June 2025, of whom 14 295 (14.2%) reported current smoking and formed our analytic sample (mean [SD] age = 42.7 [16.9]; 44.9% women; 30.5% reported current vaping; see Table S1 for a more detailed description of the sample).

**Figure 1:**
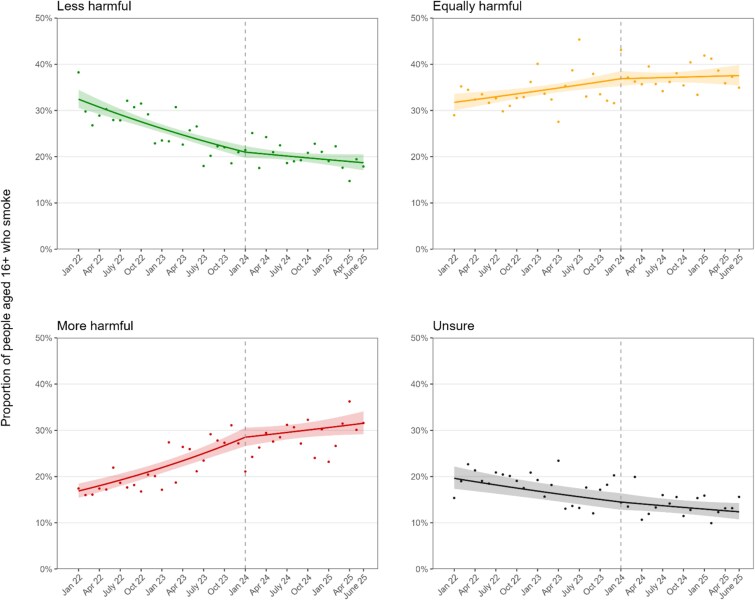
Trends in harm perceptions of e-cigarettes compared with cigarettes among people who smoke, January 2022 to June 2025. Panels show trends in the proportion of people aged ≥16 years who smoke in Great Britain who perceive e-cigarettes to be (A) less harmful than cigarettes, (B) equally harmful as cigarettes, (C) more harmful than cigarettes, and (D) those who are unsure. The vertical dashed line indicates the timing of the announcement in January 2024 of an impending ban on disposable e-cigarettes and other potential vaping restrictions. Points represent unmodeled weighted prevalence by month. Lines represent modeled weighted prevalence over the study period, adjusting for seasonality. Shaded bands represent 95% confidence intervals.

Between January 2022 and January 2024, there were substantial changes in harm perceptions of e-cigarettes among people who smoke (pre-announcement trend, Table 1 and Figure 1). The proportion who believed e-cigarettes were less harmful than cigarettes declined markedly, with a relative annual decrease of 19.6%, falling from 32.4% to 21.0%. In contrast, the proportion who believed e-cigarettes were more harmful increased by 30.0% per year, from 16.9% to 28.5%. There was also a modest annual increase of 7.8% in the belief that e-cigarettes were equally harmful and a 14.1% annual decline in the proportion who were unsure.

**Table 1:** Changes in annual trends in harm perceptions of e-cigarettes compared with cigarettes among people aged ≥16y who smoke in Great Britain since the announcement of an impending ban on disposable e-cigarettes in January 2024

	**Harm perception of e-cigarettes compared with cigarettes**			
	**Less harmful**	**Equally harmful**	**More harmful**	**Unsure**
**Annual trends, RR [95% CI]**				
Pre-announcement trend (January 22–January 24)	0.804 [0.764–0.846]	1.078 [1.033–1.124]	1.300 [1.226–1.379]	0.859 [0.805–0.917]
Change in annual trend (January 24)	1.146 [1.012–1.299]	0.940 [0.858–1.029]	0.826 [0.732–0.931]	1.042 [0.889–1.220]
Post-announcement trend (January 24–June 25)	0.922 [0.806–1.055]	1.013 [0.917–1.119]	1.073 [0.939–1.227]	0.895 [0.754–1.062]
**Predicted prevalence estimates, % [95% CI]**				
2 years pre-announcement (January 22)	32.4 [30.5–34.5]	31.8 [30.0–33.6]	16.9 [15.4–18.5]	19.6 [17.4–22.2]
1 year pre-announcement (January 23)	26.1 [25.2–27.0]	34.2 [33.3–35.2]	21.9 [20.7–23.2]	16.9 [15.2–18.7]
Month ban was announced (January 24)	21.0 [19.7–22.3]	36.9 [35.3–38.5]	28.5 [26.6–30.6]	14.5 [12.8–16.4]
1 year post-announcement (January 25)	19.3 [18.2–20.6]	37.4 [35.9–38.8]	30.6 [28.9–32.4]	13.0 [11.5–14.6]
Month ban was implemented (June 25)	18.7 [17.1–20.5]	37.5 [35.5–39.6]	31.3 [29.1–33.7]	12.5 [10.9–14.3]

Results shown are derived from segmented regression analyses (using generalized additive models) of data collected from January 2022 to June 2025. Models are adjusted for seasonality. RR = risk ratio; CI = confidence interval.

Following the government’s January 2024 announcement of an impending ban on disposable e-cigarettes and related policies, trends in harm perceptions shifted, indicating a slowing in the rate of change (Figure 1). The decline in the proportion who believed e-cigarettes were less harmful than cigarettes slowed, as did the increase in the proportion who believed e-cigarettes were more harmful (post-announcement trend, Table 1); both showed statistically significant changes in trends compared with the pre-announcement period (change in trend, Table 1). Changes in trends in the other perceptions were less certain, with 95% CIs including the possibility of no difference (change in trend, Table 1), but followed a similar pattern, suggesting relative stabilization in harm perceptions following the policy announcement.

By June 2025, when the disposable e-cigarette ban came into effect, just 18.7% of people who smoked believed e-cigarettes were less harmful than cigarettes, while 31.3% believed they were more harmful—almost twice the proportion observed in January 2022. The belief that e-cigarettes and cigarettes were equally harmful remained the most common (37.5%), and 12.5% were unsure.

Stratified analyses indicated that these overall patterns were driven primarily by people who smoked and did not vape, with no clear changes in trends observed among those who also vaped (Table S2 and Figures S1 and S2).

## Discussion

Thus far, concerns that the disposable e-cigarette ban might worsen trends in negative harm perceptions of e-cigarettes among adults who smoke are not clearly supported by the data. Since the government’s policy announcement, the proportion of adults who smoke perceiving e-cigarettes as equally or more harmful than cigarettes continued to rise; however, the pace of this increase slowed significantly compared to the period beforehand. Nevertheless, the findings highlight persistent and substantial public uncertainty and confusion regarding the harms of e-cigarettes. Stratified analyses suggested that these overall patterns were primarily driven by people who smoke but do not vape, with little evidence of post-announcement changes among those who also vape.

The pre-announcement period was marked by substantial declines in the proportion of adults who correctly perceived e-cigarettes as less harmful than cigarettes. Several factors may have contributed to this trend, including widespread negative media coverage highlighting potential risks of vaping, increasing public exposure to reports of youth uptake, and mixed messages from public health and regulatory authorities about the relative harms of e-cigarettes.^1^^,^^2^^,^^17^^,^^18^ Policy discussions and announcements prior to the ban may also have been interpreted by the public as signaling that e-cigarettes are harmful, further contributing to these shifts.

The results support a deceleration of the worsening trends in e-cigarette perceptions since the policy announcement. This may relate to the communications about vaping around the time the policy was announced: for example, the government and leading medical figures in England consistently explained that “if you smoke, vaping is much safer; if you don’t smoke, don’t vape.”^19^^,^^20^ Additionally, the announcement of substantial policies intended to reduce youth vaping may have reduced the extent of negative media coverage about vaping (e-cigarette perceptions have been shown to worsen in times of greater exposure to negative news stories about vaping^18^), but more evidence is required to clarify this. The fact that the deceleration was largely driven by people who smoke but do not vape suggests that messaging around relative harms may be particularly influential for this group. On the other hand, by the time of the announcement, harm perceptions had already deteriorated markedly. This may reflect a floor effect—where perceptions had reached such low levels that any further significant worsening was less likely. Among people who both smoked and vaped, perceptions had not reached such low levels, and trends continued to worsen in this subgroup.

Despite the deceleration in the deterioration of perceptions, the most recent prevalence estimates highlight persistent and substantial public uncertainty and confusion regarding the harms of e-cigarettes and underscore the need for clear, evidence-based communication to improve public understanding. Harm perceptions play a significant role in influencing decisions around e-cigarette use and smoking cessation.^5^ Evidence suggests that viewing e-cigarettes as equally or more harmful as cigarettes—or being unsure about their relative harms—can discourage people who smoke from using e-cigarettes as a harm reduction tool and increase relapse to smoking^5^ despite robust evidence indicating that e-cigarettes are both less harmful^1^ and effective in supporting smoking cessation.^21^

These findings have several important implications. First, policymakers should consider that public perceptions of the relative harms of e-cigarettes are already poor—particularly among people who smoke but do not vape—and that increased regulation could potentially be interpreted as suggesting that e-cigarettes are as, or more, harmful than smoking. Clear, evidence-based messaging alongside regulatory actions is therefore important to help prevent unintended negative impacts on harm perceptions and smoking cessation behaviors. Second, public health campaigns should prioritize education about the relative risks of different nicotine products, emphasizing that switching from smoking to vaping can reduce harm. Third, healthcare providers can play a key role in reinforcing accurate perceptions during smoking cessation interventions, particularly among adults who smoke and may be uncertain or misinformed about e-cigarette harms. Future research should monitor long-term changes in harm perceptions and vaping behaviors following new policy implementations in England as well as other countries, including how trends differ between people who smoke who do and do not vape, to understand how perceptions evolve over time.

Several limitations should be noted. The observational nature of the data limits causal inference regarding the policy announcement’s direct impact on harm perceptions. Additionally, the relatively short post-announcement period may not capture longer-term changes in perceptions or behaviors—especially following the implementation of the ban. The sample also only comprised adults who smoke and estimates of accurate harm perceptions are lower than adults who smoke in other national surveys, potentially due to the sampling or survey methods (eg, online vs. telephone interviews).^3^ Telephone interviews may introduce social desirability or interviewer effects, whereas online surveys may allow respondents to answer more anonymously, potentially affecting reported perceptions.

Ongoing monitoring of harm perceptions and behaviors remains essential to evaluate the longer-term effects of new vaping policies and to guide effective communication strategies that promote informed decision-making and support harm reduction.

## Supplementary Material

Supplementary_File_ntag030

## Data Availability

Data used for these analyses are available on Open Science Framework (https://osf.io/vrkua/).
