# Supplementary material for: Changes in harm perceptions of e-cigarettes compared with cigarettes following the announcement of the disposable vape ban in Great Britain
Source: Nicotine Tob Res. 2026 Feb 12;28(8):1443–8. doi: 10.1093/ntr/ntag030 (PMC13389525; doi:10.1093/ntr/ntag030)
Supplement: Supplementary_File_ntag030 [file supplementary_file_ntag030.docx]

**Table S1.** Weighted sample characteristics (*n*=14,295)

|  | **%**^1^ |
| --- | --- |
|  |  |
| Age |  |
| Mean (SD) | 42.7 (16.9) |
| 16-24 | 16.8 |
| 25-34 | 23.1 |
| 35-44 | 17.9 |
| 45-54 | 16.3 |
| 55-64 | 13.4 |
| 65+ | 12.6 |
|  |  |
| Gender |  |
| Man | 53.8 |
| Woman | 44.9 |
| Other | 1.3 |
|  |  |
| Current vaping |  |
| Yes | 30.5 |
| No | 69.5 |
|  |  |

^1^ Unless otherwise specified.

**Table S2.** Changes in annual trends in harm perceptions of e-cigarettes compared with cigarettes among people aged ≥16y who smoke in Great Britain since the announcement of an impending ban on disposable e-cigarettes in January 2024 – stratified by vaping status

|  | **Harm perception of e-cigarettes compared with cigarettes** | | | | |
| --- | --- | --- | --- | --- | --- |
|  | **Less harmful** | **Equally harmful** | **More harmful** | **Unsure** | |
|  |  |  |  |  | |
| **People who smoke and do not vape (unweighted *n*=10,173)** |  |  |  |  | |
|  |  |  |  |  | |
| **Annual trends, RR [95% CI]** |  |  |  |  | |
| Pre-announcement trend (Jan 22 – Jan 24) | 0.698 [0.648-0.752] | 1.067 [1.015-1.121] | 1.333 [1.253-1.418] | 0.867 [0.809-0.930] | |
| Change in annual trend (Jan 24) | 1.471 [1.217-1.777] | 0.934 [0.836-1.043] | 0.778 [0.686-0.882] | 0.998 [0.840-1.185] | |
| Post-announcement trend (Jan 24 – June 25) | 1.026 [0.838-1.258] | 0.996 [0.882-1.124] | 1.037 [0.902-1.193] | 0.865 [0.719-1.042] | |
|  |  |  |  |  | |
| **Predicted prevalence estimates, % [95% CI]** |  |  |  |  | |
| 2 years pre-announcement (Jan 22) | 24.3 [21.1-27.9] | 33.1 [30.5-35.9] | 19.3 [17.6-21.2] | 22.1 [19.4-25.2] | |
| 1 year pre-announcement (Jan 23) | 16.9 [15.0-19.1] | 35.3 [33.2-37.5] | 25.7 [24.3-27.2] | 19.2 [17.2-21.4] | |
| Month ban was announced (Jan 24) | 11.8 [10.2-13.7] | 37.7 [34.9-40.6] | 34.3 [32.0-36.8] | 16.6 [14.6-18.9] | |
| 1 year post-announcement (Jan 25) | 12.1 [10.6-13.9] | 37.5 [35.1-40.1] | 35.6 [33.5-37.7] | 14.4 [12.7-16.3] | |
| Month ban was implemented (June 25) | 12.3 [10.4-14.5] | 37.4 [34.6-40.6] | 36.0 [33.4-38.9] | 13.7 [11.8-15.9] | |
|  |  |  |  |  | |
| **People who smoke and vape  (unweighted *n*=4,122)** |  |  |  |  | |
|  |  |  |  |  | |
| **Annual trends, RR [95% CI]** |  |  |  |  | |
| Pre-announcement trend (Jan 22 – Jan 24) | 0.874 [0.816-0.937] | 1.110 [1.023-1.205] | 1.259 [1.071-1.479] | 0.898 [0.758-1.064] | |
| Change in annual trend (Jan 24) | 0.990 [0.838-1.170] | 0.935 [0.788-1.109] | 0.986 [0.714-1.359] | 1.116 [0.763-1.631] | |
| Post-announcement trend (Jan 24 – June 25) | 0.866 [0.723-1.037] | 1.038 [0.859-1.254] | 1.241 [0.866-1.778] | 1.002 [0.661-1.519] | |
|  |  |  |  |  | |
| **Predicted prevalence estimates, % [95% CI]** |  |  |  |  | |
| 2 years pre-announcement (Jan 22) | 50.5 [45.0-56.6] | 29.7 [25.9-33.9] | 8.9 [6.6-12.1] | 11.7 [9.3-14.6] | |
| 1 year pre-announcement (Jan 23) | 44.1 [40.3-48.3] | 32.9 [30.0-36.2] | 11.2 [8.9-14.2] | 10.5 [9.3-11.8] | |
| Month ban was announced (Jan 24) | 38.6 [34.4-43.2] | 36.6 [32.6-41.0] | 14.2 [10.9-18.4] | 9.4 [7.8-11.4] | |
| 1 year post-announcement (Jan 25) | 33.4 [29.9-37.2] | 38.0 [34.3-42.0] | 17.6 [14.0-22.0] | 9.4 [7.9-11.2] | |
| Month ban was implemented (June 25) | 31.4 [27.3-36.1] | 38.4 [34.1-43.4] | 18.9 [14.7-24.1] | 9.5 [7.4-12.0] | |
|  |  |  |  |  | |
| RR, risk ratio; CI, confidence interval.  Results shown are derived from segmented regression analyses (using generalised additive models) of data collected from January 2022 to June 2025. Models are adjusted for seasonality. | | | | |  |

#####
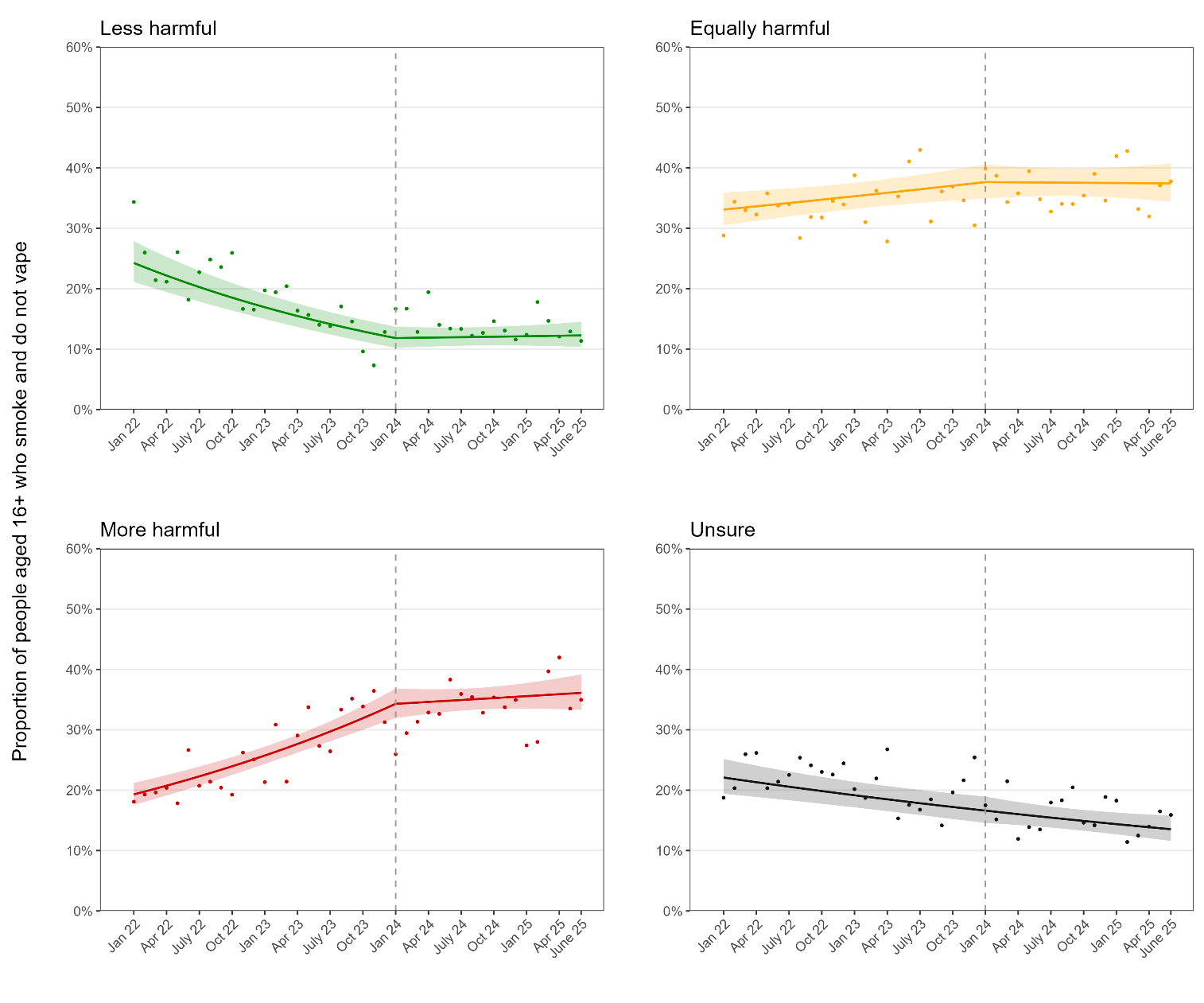


##### **Figure S1. Trends in harm perceptions of e-cigarettes compared with cigarettes among people who smoke and do not vape, January 2022 to June 2025.**

Panels show trends in the proportion of people aged ≥16y who smoke and do not vape in Great Britain who perceive e-cigarettes to be (a) less harmful than cigarettes, (b) equally harmful as cigarettes, (c) more harmful than cigarettes, and (d) those who are unsure. The vertical dashed line indicates the timing of the announcement in January 2024 of an impending ban on disposable e-cigarettes and other potential vaping restrictions. Points represent unmodelled weighted prevalence by month. Lines represent modelled weighted prevalence over the study period, adjusting for seasonality. Shaded bands represent 95% confidence intervals.

#####
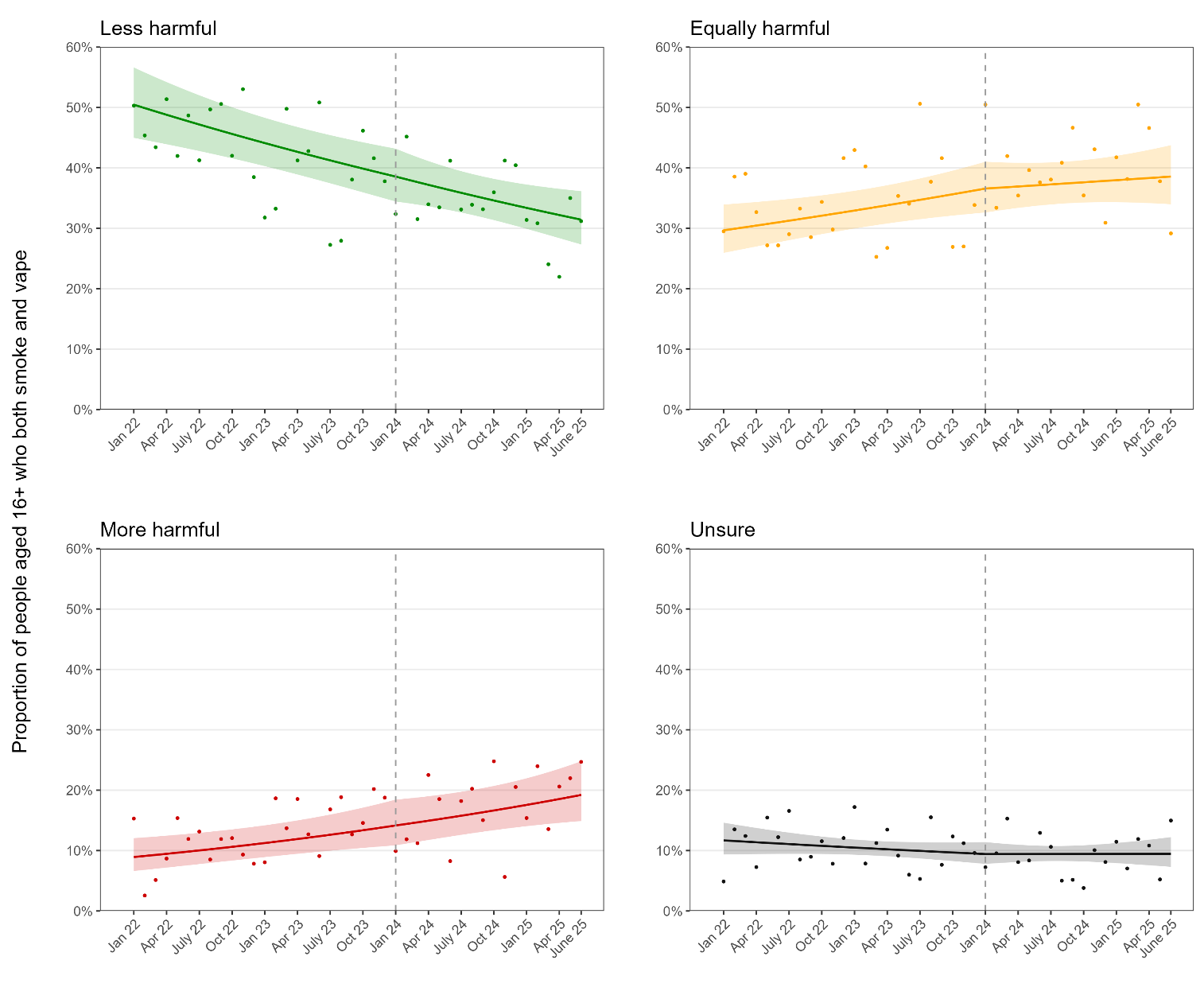


##### **Figure S2. Trends in harm perceptions of e-cigarettes compared with cigarettes among people who both smoke and vape, January 2022 to June 2025.**

Panels show trends in the proportion of people aged ≥16y who both smoke and vape in Great Britain who perceive e-cigarettes to be (a) less harmful than cigarettes, (b) equally harmful as cigarettes, (c) more harmful than cigarettes, and (d) those who are unsure. The vertical dashed line indicates the timing of the announcement in January 2024 of an impending ban on disposable e-cigarettes and other potential vaping restrictions. Points represent unmodelled weighted prevalence by month. Lines represent modelled weighted prevalence over the study period, adjusting for seasonality. Shaded bands represent 95% confidence intervals.
